# FishSizer: Software solution for efficiently measuring larval fish size

**DOI:** 10.1002/ece3.8672

**Published:** 2022-03-06

**Authors:** Jeppe Have Rasmussen, Marta Moyano, Lee A. Fuiman, Rebekah A. Oomen

**Affiliations:** ^1^ 4343 Center for Coastal Research University of Agder Kristiansand Norway; ^2^ 4343 Center for Artificial Intelligence Research University of Agder Kristiansand Norway; ^3^ Marine Science Institute University of Texas at Austin Port Aransas Texas USA; ^4^ Center for Ecological and Evolutionary Synthesis University of Oslo Oslo Norway

**Keywords:** body size, early life stage, growth, larvae, morphometrics, open‐source software

## Abstract

1. Length and depth of fish larvae are part of the fundamental measurements in many marine ecology studies involving early fish life history. Until now, obtaining these measurements has required intensive manual labor and the risk of inter‐ and intra‐observer variability.
2. We developed an open‐source software solution to semi‐automate the measurement process and thereby reduce both time consumption and technical variability. Using contrast‐based edge detection, the software segments images of a fish larva into “larva” and “background.” Length and depth are extracted from the “larva” segmentation while taking curvature of the larva into consideration. The graphical user interface optimizes workflow and ease of usage, thereby reducing time consumption for both training and analysis. The software allows for visual verification of all measurements.
3. A comparison of measurement methods on a set of larva images showed that this software reduces measurement time by 66%–78% relative to commonly used software.
4. Using this software instead of the commonly used manual approach has the potential to save researchers from many hours of monotonous work. No adjustment was necessary for 89% of the images regarding length (70% for depth). Hence, the only workload on most images was the visual inspection. As the visual inspection and manual dimension extraction works in the same way as currently used software, we expect no loss in accuracy.

## Graphical abstract

Measuring larval fish is important in many ecological research project. Up to now it has been very time‐consuming manual work. We have created a workflow optimized semi‐automated software solution for this task.

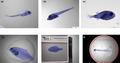

## 1 INTRODUCTION

Larval growth rate is a fundamentally important life history trait directly linked to fish population productivity and persistence. For many fish populations, fast‐growing larvae have higher survival rates, largely by reducing larval stage duration in which they are exposed to higher predation risk (Houde, 2008). Also, larger body sizes are associated with increased feeding and swimming capacity of larvae, allowing them to search larger water volumes, increase encounter rates with prey, feed on larger organisms, and have better escape responses from predators (Hale, 1996; Miller et al., 1988; Munk & Kiorboe, 1985; Peck et al., 2012). Therefore, body length is an essential measurement in studies of larval fish ecology (Bils et al., 2017), development (Fuiman et al., 1998), physiology (Illing et al., 2016; Petereit et al., 2008), evolution (Oomen & Hutchings, 2015, 2016; Oomen et al., 2021), reproductive biology (Roney et al., 2018), and aquaculture (Blanco et al., 2017). Besides length, body depth is also valuable, as it can be used as a proxy for muscle development (i.e., myomere height) and potential starvation resistance (Moyano et al., 2016; Peña et al., 2021).

Body size measurements of larval fish are generally taken on individuals using a camera attached to a stereomicroscope. On the generated images, body length and depth are measured manually using a dedicated software, either open‐source (e.g., ImageJ) or proprietary (e.g., Leica Application Suite). Body length is generally estimated as notochord length (tip of the snout to the end of the notochord) during the preflexion stage and as standard length (tip of the snout to the posterior extremity of the hypural plate) afterwards (Kahn et al., 2004). Body depth is generally measured as maximum depth at the head, at the anus, or at the caudal peduncle. Even with the aid of dedicated software, these manual measurements are time consuming because a typical laboratory study can generate thousands of images (e.g., 4489 by Roney et al. (2018)). This manual work also has high potential for introducing intra‐ and inter‐observer variability, potentially leading to increased measurement errors.

Here, we introduce a novel, open‐source application, FishSizer, to semi‐automate measurements of larval fish length and myomere depth. FishSizer addresses the two major problems of the manual method used to estimate body size in larval fish by considerably reducing the amount of manual work and potentially decreasing technical variability. Although this application was developed specifically for use with fish larvae, it should be useful for older life stages of fish (and possibly other animals) for which satisfactory images are available.

## 2 COMPUTATIONAL BACKGROUND

### 2.1 Overall method

All coding was done in Matlab 2020a (The MathWorks Inc.) and compiled into a single file for installation on computers without Matlab. Determination of length and depth of fish larvae from images is based on A sequence of two procedures: (1) producing a mask (a binary image the same size as the original image) segmented into “larva” and “background” and (2) determining length and depth based on this “larva” segmentation.

### 2.2 Segmentation

For morphometrics assessments, pictures of anesthetized individual larvae laying on a microscope slide are taken under a stereomicroscope. During a single session, a user may typically take hundreds of pictures of individual larvae, so the zoom level is typically fixed in order to reduce the manipulation time. This procedure results in the background being mostly uniform (i.e., low in contrast) with the exception of lines arising from scratches on the glass slide and water drops. Due to this low‐contrast background, segmentation is based on edge detection. Edge detection is a method of establishing regions where the contrast between neighboring pixels in an image is above a certain preset threshold. Edge detection has the advantage of being applicable across species and/or stage of the larvae being measured. More advanced methods, such as deep learning, will in most cases need retraining of the network to correctly segment species not previously encountered by the network (Kvæstad et al., 2022). Deep learning has the additional drawback of having higher hardware demands compared to this less computationally intensive approach (LeCun, 2019). In order to determine a robust edge detection threshold across a wide range of images, we determine the maximum contrast present in the image and set the threshold as a customizable fraction of this value. Many images used for larval fish length measurements contain scale bars or other high‐contrast objects. To avoid basing the contrast threshold on these artifacts, this software offers the option of establishing a region of interest (ROI) within which the segmentation is contained.

**FIGURE 1:**
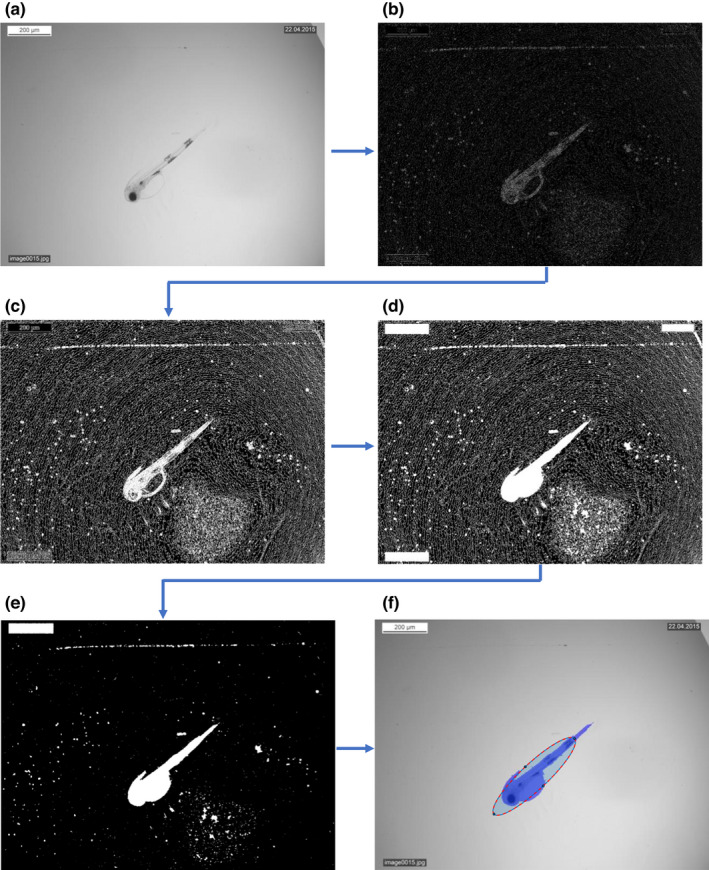
Segmentation process illustrated. (a) Original image; (b) Edge detection on Gaussian filtered image; (c) Dilation of all edges followed by erosion; (d) Filling in all enclosed areas; (e) Keeping only areas, removing lines; (f) Keep only largest area and extraction orientation (segmentation superimposed on original image for comparison)

**FIGURE 2:**
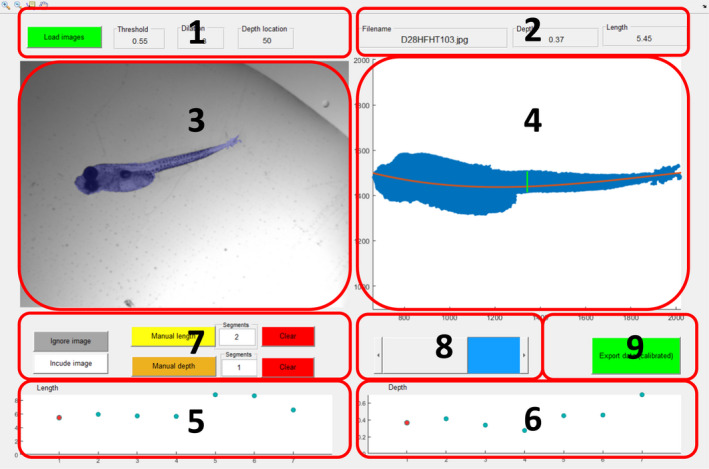
Graphical user interface (1) Panel associated with loading and segmentation settings. (2) Information about the active image. (3) Top left window displaying original image in black and white with segmentation mask overlayed in blue. (4) Top right window displaying zoomed in larva segmentation with length and depth estimation locations marked. (5) Bottom left window giving overview of length estimations for all images for easy outlier detection. (6) Bottom right window giving overview of depth estimation for all images for easy outlier detection. (7) Manual measurement panel. (8) Slider for navigating between images. (9) Export data button for extracting data into CSV file

Running the edge detection algorithm on an image creates a binary mask with pixel values of 1 at edges and 0 at other locations (see Figure 1 for the segmentation process illustrated). The aim is to have a complete outline of the larva and subsequentially fill in this outline. In practice, the detected outline can be incomplete, leaving small gaps and resulting in faulty segmentation. We therefore dilate all detected edge pixels by a customizable factor (default = 3), which can result in exaggerated edges. These artifacts are compensated for using image erosion, a process of setting all pixel values within a morphological structuring element (in this case a diamond) to the minimum values within said element. After erosion, all areas within the mask that are surrounded by edges are then filled in. The larva must be completely visible in the image and not touching any edge of the image. All areas connected to an image border are ignored. This is done because there often are drops of water across the picture, creating a high‐contrast line from one point on the edge of the image to another (first image example in the manual shows a typical situation). The largest segment is selected as the larva and remaining segments are collectively labeled as background. For visual verification of the segmentation, the original image with the corresponding semi‐transparent mask overlaid is shown in the graphical user interface (Figure 2, top left window). For further analysis, we extract orientation for an ellipse that best represents this region.

**FIGURE 3:**
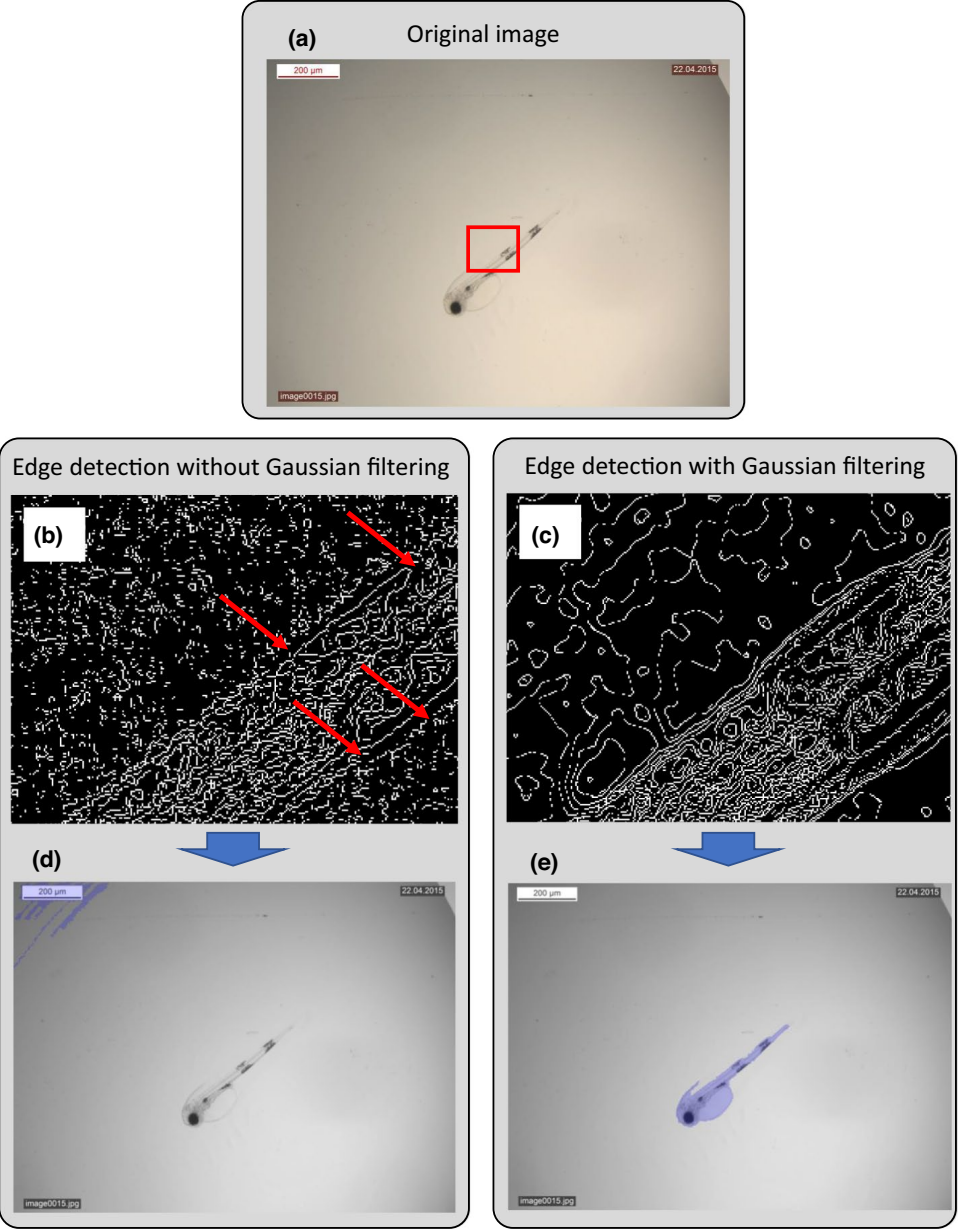
Example of difference in segmentation with and without Gaussian image filtering. (a) Original image. Red square marks area shown in (b) and (c). (b) Zoomed image of edge detection without Gaussian image filtering (after dilation). Red arrows point to gaps in the outline of the larva; (c) zoomed in image of edge detection with Gaussian image filtering (after dilation). Outline of the larva is uninterrupted; (d) resulting segmentation without Gaussian image filtering. As the outline of larvae was incomplete, the software failed to segment the larva but instead segmented the text box in the upper left corner. (e) Resulting segmentation from using Gaussian image filtering. As there were no gaps in the outline of the larva, segmentation was correct

In order to minimize the need to adjust parameter settings, and make the software as user friendly as possible, we aimed to make the segmentation as robust as possible. We found that a Gaussian image filter with a standard deviation of four pixels followed by an image sharpening algorithm with the same standard deviation significantly improved segmentation (e.g., Figure 3). When applying a Gaussian image filter, the value of each pixel is influenced by the value of all neighboring pixels. Hence, small regions in the outline with low contrast can have increased contrast after the procedure and, therefore, a better chance of complete edge detection.

### 2.3 Length and depth measurement

Length and depth estimation is done using the segmentation mask. The first step is to rotate the mask using the orientation of the major axis of the ellipse fitted to the larva outline. This rotation is done using the imrotate.m function in MATLAB. By default, imrotate uses nearest‐neighbor interpolation, setting the values of pixels in the final image that are outside the rotated image to 0. Alignment with the horizontal (X) axis allows the use of a polynomial regression for length estimation. It also allows depth estimation based solely on the Y component at a customizable location relative to the length (X axis). The next step is to establish which end is the head end by dividing the larva into two halves of equal length and determining which end contains more pixels in the segmented mask. This procedure assumes that the anterior half of the larva is larger than the posterior half.

Length estimation is based on a polynomial regression line through the larva segmentation that best describes the curvature of the larva. As larval fish are often curved, we use second‐order or greater polynomial regression. Presence of a large yolk sac affects the regression, such that a larva with straight notochord and large yolk sac will yield a curved regression that does not accurately represent the larva's length. To avoid this, we base the order of the regression on the curvature of the tail alone. The greater the curvature, the higher the order of the polynomial regression. A lower‐order regression on a larva predicted to be straight will result in a straighter regression and hence a more precise length estimation. Therefore, a second‐order polynomial regression is computed for the tail part of the larva and, since the larva is rotated to be horizontal, both coefficients of this regression indicate the amount of curvature of the entire larva. If the absolute value of the second‐degree coefficient is >0.1 or the absolute value of the first‐degree coefficient is >0.5, a third‐order polynomial regression is used for the entire larva. Otherwise, a second‐order polynomial regression is used.

Depth is determined at a customizable location relative to the length of the larva (X axis in Figure 2, window 2). Because the segmented larvae are rotated to be horizontal, a good approximation of the depth is the difference between the maximum and minimum Y values at the user‐defined X location. To make depth determination less dependent on precise point‐to‐point segmentation, depth is not calculated at a single point but rather as the median of all depths calculated at the user‐defined location ±5% of the length of the larva. For visual verification, length and depth estimation lines are shown in the main graphical user interface (Figure 2 window 2).

## 3 PROGRAM DESCRIPTION

### 3.1 Main GUI window description

The interface of this software is split into two main graphical user interfaces: the main GUI and the loading GUI. The main graphical user interface contains four windows and five button panels as seen in Figure 2. Top left window shows the original image overlaid with a semi‐transparent larva segmentation mask for visual confirmation of correct segmentation. Top right window shows the rotated segmentation mask with length and depth estimation lines overlaid, allowing for verification of correct placement of the two measurements. Bottom left window displays the length estimates for all images (represented as circles), to facilitate outlier detection. The active image shown in windows 1 and 2 is color‐coded red. The software allows for manual estimation of length and depth if the automated estimation is unsatisfactory. If manual estimation is used, the marker representing the image is changed from a circle to a diamond. Bottom right window shows the depth estimation for all images and uses the same marker symbolism as window 3.

### 3.2 Main GUI button description

Buttons in the GUI are grouped according to function (Figure 2). Icons for zooming top left window are in the top left corner. The magnifying glass with a “+” symbol zooms in. The magnifying glass with a “−“ symbol zooms out. The notepad with crosshairs displays values for the selected pixel. The “hand” symbol moves the zoomed image. The button for opening the loading GUI is located above the top left window, as are settings for segmentation. See section 3.1.2 and the FishSizer manual for details.

Above the top right window are three displays corresponding to (1) the filename of the active image, (2) the depth estimate, and (3) the length estimate. The values are displayed in millimeters (mm) if a calibration has been performed (see section 3.3).

Below the top left window are buttons for manual measurements. To the left are buttons for excluding the active image from a dataset if its quality is insufficient: one for excluding the image and one for reversing the action. “Manual length” and “Manual depth” buttons allow the user to manually draw lines in top left window to measure length and depth. To the right of these two buttons is a panel for setting the number of line segments needed to make the measurement. The default for measuring length is 2, as most larvae are slightly curved and hence a line consisting of two segments will give a more accurate estimate of the length than one straight line. If the number of segments is set greater than needed for a particular larva, pressing “enter” after marking fewer segments will complete the measurement. The default number of segments for manually measuring depth is 1, which usually is sufficient. To the right are buttons for cancelling the manual length or depth estimations and reverting to the automated estimations.

Below the top right window are controls for navigating the image files and exporting data to a comma‐delimited file (.csv). To the left is a slider for selecting images from the loaded dataset. For optimized workflow, hotkey functions are associated with this. Pressing “a” selects the image prior to the active image, pressing “s” selects the image after the active image. To the right is the export data button. Data can be exported both with and without calibration into millimeters (mm). The “export data” button is yellow if data have not been calibrated (measurement reported in units of pixels) and green if calibration into millimeters (mm) has been applied to the dataset.

#### 3.2.1 Loading GUI description

**FIGURE 4:**
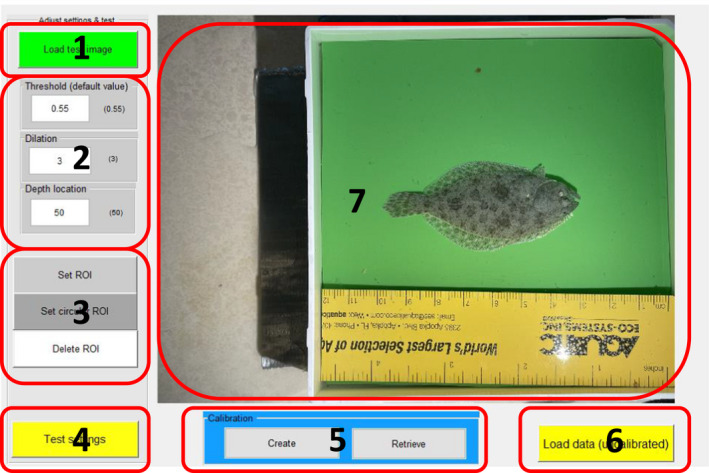
Loading GUI. (1) Load test image. (2) Set segmentation settings and location for depth measurement. (3) Set rectangular or circular ROI for segmentation. (4) Test settings and ROI. (5) Calibrate pixel measurements into millimeters. (6) Load dataset using settings displayed in Panel 2. (7) Window displaying effect of settings on test image

This GUI provides the options to (1) load a test image for testing and adjusting the segmentation settings, and (2) establish a region of interest (Figure 4). “Load test image” opens a dialog box allowing the user to select a typical image from the dataset. The settings panel below the “Load test” button contains three buttons: “Threshold” sets the fraction of maximum contrast (see section 2.2). “Dilation” sets the number of dilations applied to each edge‐detected pixel (see section 2.2).” Depth offset” determines the location of the depth measurement in percentage of total larval length from the head. If set to 50, depth is measured at the midpoint of the larva along the X axis in window 2 of the main GUI.

The ROI panel contains three buttons for: (1) setting a rectangular ROI, (2) setting a circular ROI, and (3) cancelling a set ROI. The “Test settings” button processes the test image using the current setting for segmentation and ROI and displays the result for visual inspection. A calibration panel below the central image panel contains two buttons for: (1) creating a new calibration file, and (2) retrieving a previously created calibration file. These calibration files change the dimensions from pixels to millimeter. Pressing the Create Calibration button opens up a new GUI for marking a known distance on an image that contains a visible scale (see the manual for details). The Retrieve buttons allow for loading calibration files previously created via the Create Calibration button.

### 3.3 Workflow

A typical workflow with a set of images is first to confirm or change the segmentation settings on the loading GUI. It is recommended to load a test image and try settings before loading the entire dataset. By visually inspecting segmentation on images of Atlantic cod (*Gadus morhua*), Atlantic bluefin tuna (*Thunnus thynnus*), Pufferfish (*Tetraodontidae* spp.), European plaice (*Pleuronectes platessa*), Southern flounder (*Paralichthys lethostigma*), and Atlantic herring (*Clupea harengus*) generated by different users, we found a default values of 0.55 for threshold and 3 for dilation to be appropriate. Lowering the threshold can be helpful if not enough of the larva is segmented and the background contains little contrast. Optimal depth location depends on the species of fish being researched. For Atlantic cod (*Gadus morhua*) and Atlantic bluefin tuna (*Thunnus thynnus*), the default of 50 achieved good results, meaning that depth was measured at the midpoint of the length of the larva. Calibration of pixel‐based measurements into millimeters is done via the calibration panel, either by retrieving a previously created calibration file or by creating a new one (see manual for details). After calibration, the “Load dataset” button is green to reflect that calibration has been done. After visual validation of correct segmentation settings, images are loaded with the “Load images” button, after which a progress bar appears. When the progress bar disappears, the slider below window 2 in the main GUI, as well as hotkeys “a” and “s”, can be used to navigate the dataset. If automatic estimation is satisfactory, no additional work is needed, and the user can move to the next image. If the automatic estimation is unsatisfactory, manual length and depth extraction is done in the same way as ImageJ. The “Export data” button is green if calibration has been performed and pressing the button will produce a comma‐delimited file with five (without calibration) or nine (with calibration) columns containing data as described in Table 1.

**TABLE 1:** Data in exported file

Column number	Name	Description
1	FileName	Name of image file
2	Length_px	Length in pixels. If no manual measurement was done, automated estimation is used
3	LengthAutomated_px	Automated length estimation in pixels
4	Depth_px	Depth in pixels. If no manual measurement was done, automated estimation is used
5	DepthAutomated_px	Automated depth estimation in pixels
6	Length_mm	Length in mm. If no manual length was done, automated is used
7	LengthAutomated_mm	Automated length estimation in mm
8	Depth_mm	Depth in mm. If no manual measurement was done, automated estimation is used
9	DepthAutomated_mm	Automated depth estimation in mm

Red columns are present only when data calibration has been performed.

## 4 PERFORMANCE EVALUATION

**FIGURE 5:**
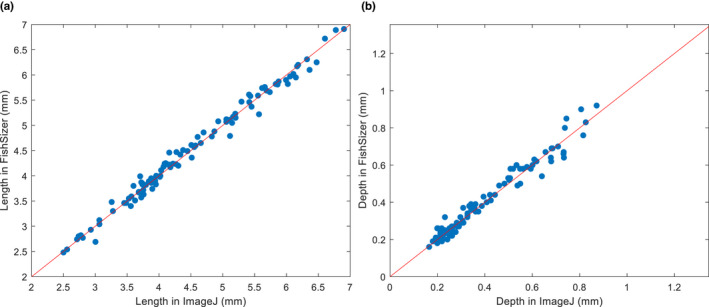
Length (a) and depth (b) from 101 haphazardly selected images of tuna larvae representing various developmental stages measured in imageJ versus FishSizer. For both dimensions, a paired t test shows no difference in the measurements on a 5% significance level (Length: *p *= .49; depth *p *= .27)

Performance of this software depends heavily on the quality of the images used. We tested it on a set of 101 haphazardly chosen tuna larva images. Here, the software estimated the length correctly (±5% of total larva length) in 89% and depth in 70% of the images. A comparison of the length and depth values extracted via the semi‐automated method of FishSizer with the manual measurements from ImageJ showed strong agreement throughout the range of sizes measured (Figure 5). A paired t test (α = .05) showed no difference in the measurements (length: *p* = .49, depth *p* = .27). Time savings were calculated compared to ImageJ (Schneider et al., 2012), a commonly used software for this task. Two independent observers went through the test dataset and extracted length and depth in FishSizer, including visually verifying all measurements. Compared to extracting the same measurements from the same dataset in ImageJ, a time saving of 66 and 78% was observed for observer 1 (R1) and 2 (R2), respectively. Intra‐observer variability is expected to be lower using FishSizer compared to ImageJ as the threshold for accepting automatic parameter extraction will remain fairly constant for each observer. For manually extracted parameters in FishSizer, variability is expected to be the same as ImageJ, as the process for manual extraction is the same in both software packages.

**FIGURE 6:**
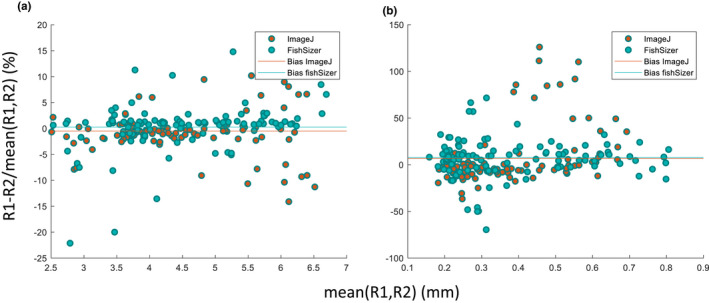
Bland–Altman plot for inter‐observer variability for 101 haphazardly chosen tuna larva images. Y axes show percentage deviation between two observers, R1 and R2. X axes show mean of the two observations. (a) Plot for length and (b) Plot for depth

To examine inter‐observer variability compared to ImageJ, we analyzed data from two observers to compare percentage‐wise differences in length and depth measurements across 101 haphazardly chosen tuna larva images (Figure 6). For length we found a deviation between the two observers of 2.69%±3.35% (mean±std) for FishSizer compared to 3.13% ± 4.54% for ImageJ. For depth we found a mean of 15.6% ± 14.6% for FishSizer and 19.8% ± 34.0% for ImageJ. Therefore, there were a small but not significant difference between the two software packages for both parameters. For visual representation of the variability using a Bland–Altman graph, see Figure 6. One important aspect of variability is human error. In FishSizer, all handling of parameters and data output is automatic and linked to the image file name. Therefore, FishSizer has less potential to introduce human errors than other programs which can require manual data curation (e.g., using copy/paste), such as ImageJ.

## 5 CONCLUSION AND FUTURE DIRECTIONS

The partial automation of obtaining larval morphological measurements in this software saves a considerable amount of time compared to the manual procedure used so far. Even when all measurements obtained via our software were manually verified and corrected, we experienced a time saving of 66%–78%.

**FIGURE 7:**
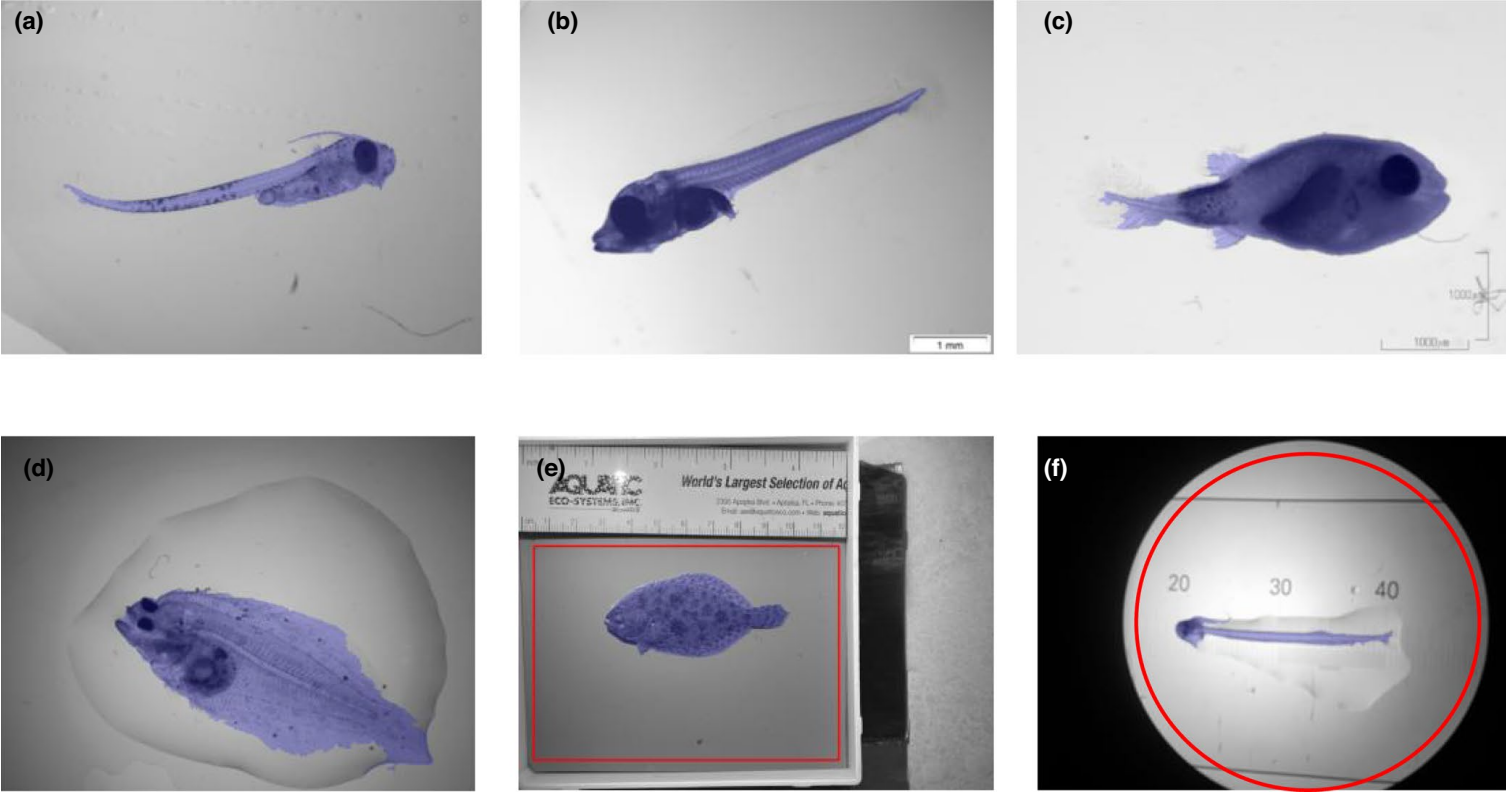
Examples of correct segmentation across species and stages. (a) Atlantic cod (*Gadus morhua*), (b) Atlantic bluefin tuna (*Thunnus thynnus*), (c) Pufferfish (Tetraodontidae spp.), (d) European plaice (*Pleuronectes platessa*), and (e) Southern flounder (*Paralichthys lethostigma*) using a rectangular ROI, and (f) Atlantic herring (*Clupea harengus*) using a circular ROI

**FIGURE 8:**
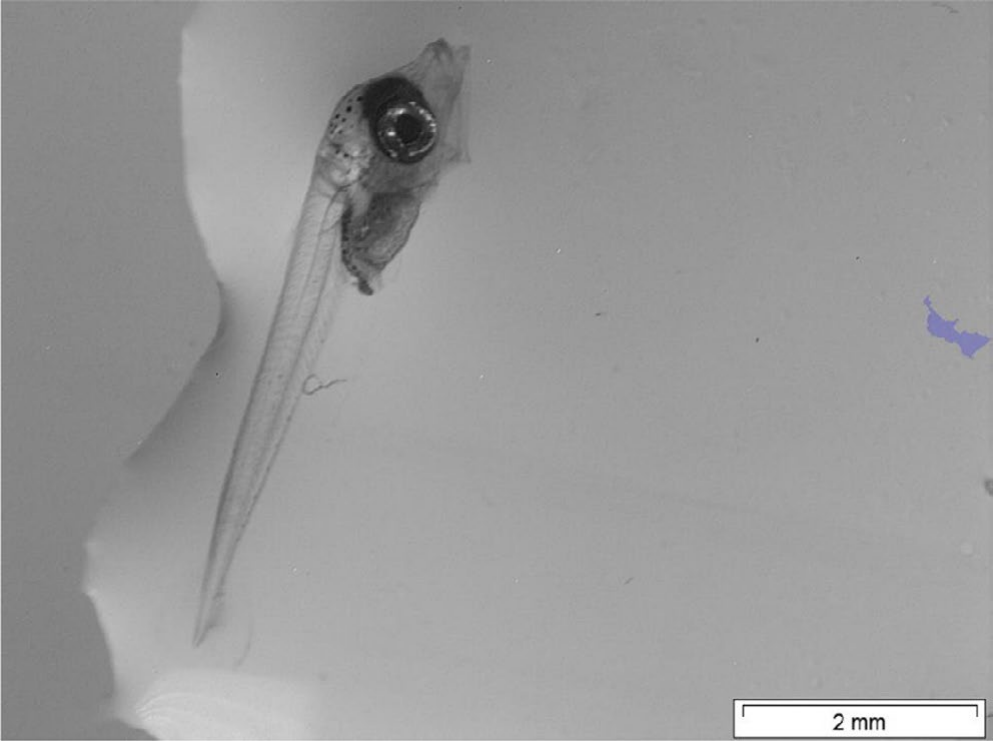
Example of faulty segmentation of an Atlantic bluefin tuna larva. In this case, the strong contrast of the water edge to the left is connected to the contrast associated with the larva itself. As all areas directly connected to the borders of the image are ignored, a small area to the right is chosen as largest area surrounded by a complete outline

When tested across a range of species, we found the software to achieve good segmentation for a wide range of species like Atlantic cod, Atlantic bluefin tuna, Pufferfish, European plaice, and Southern flounder using a rectangular ROI, and Atlantic herring using a circular ROI. (Figure 7). As the parameter extraction relies heavily on segmentation, we expect that the accuracy for these and similar species will be the same as seen for the tuna statistically investigated here. Unfortunately, as the software is based on contrast detection, we found the quality of images to be essential. High‐contrast artefacts in direct contact with the larva can hinder correct segmentation and thereby correct parameter extraction (Figure 8).

A logical future direction for this software will be to include deep learning. This current version of FishSizer can help create large datasets of segmented larva images to be used as ground truth for training neural networks. Deep learning will not only facilitate analysis of images with more than one larva, a sought after feature for this kind of software, but it will also open the door to automatic species and/or stage identification of larval fish, which is far behind other pelagic marine organisms such as plankton (Guo et al., 2021).

## CONFLICT OF INTEREST

All authors declare no conflict of interest.

## AUTHOR CONTRIBUTIONS

**Jeppe Have Rasmussen:** Conceptualization (equal); Data curation (equal); Methodology (equal); Project administration (equal); Software (lead); Validation (equal); Visualization (equal); Writing – original draft (lead); Writing – review & editing (equal). **Marta Moyano:** Conceptualization (equal); Data curation (equal); Formal analysis (equal); Methodology (equal); Validation (equal); Writing – review & editing (equal). **Lee A. Fuiman:** Methodology (equal); Validation (equal); Writing – review & editing (equal). **Rebekah Alice Oomen:** Conceptualization (equal); Formal analysis (equal); Funding acquisition (equal); Methodology (equal); Validation (equal); Writing – review & editing (equal).

## Supporting information

Appendix S1

## Data Availability

Compiled installable software, manual, and MATLAB files used for compiling are available at: https://doi.org/10.5281/zenodo.5833209 (https://github.com/jeppehave/FishSizer).
