# Supplementary material for: FishSizer: Software solution for efficiently measuring larval fish size
Source: Ecol Evol. 2022 Mar 6;12(3):e8672. doi: 10.1002/ece3.8672 (PMC8928902; doi:10.1002/ece3.8672)
Supplement: Supplementary file 1 — Appendix S1 [file ECE3-12-e8672-s001.docx]

Appendix S1: Manual for FishSizer V3

# Requirements

This version of FishSizer works only on **Windows computers**. To install the app, please run the installation file. The program does not need any other app installed to work, but during first installation a free version of Matlab runtime will be installed. This is a large file so expect significant loading time.

Only **one larva per image** is detected in this version. We aim to release a version able to measure multiple larvae per image at a later point.

Calibration of pixels into millimeters is done manually for sets of images, hence this software will save most time if all, or sets of, the images have been taken at a **fixed zoom level**.

This software is based on contrast detection. After detecting pixels with large contrast to neighboring pixels, a dilation (followed by erosion) is done to connect these pixels to create a complete outline of the larva. All uninterrupted outlines are then filled in and only the largest outline is kept. Hence, segmentation of the larva will work best if there is **little to no contrast within the background**. In cases where the contrast is present in predictable areas of the image (e.g., a measurement scale), the designation of a region of interest (ROI) can help the software ignore those regions

For optimal results, we suggest using jpg or tiff files with a **resolution of 2 megapixels** or more.

On a midlevel laptop the software uses around **3 seconds to process each image**. Images containing many irrelevant high contrast areas will increase processing time. The use of ROI is recommended in these cases.

Transparent fins will demand very low background contrast combined with a low threshold setting to be detected.

# Image examples

The following images provide examples of good and bad images:


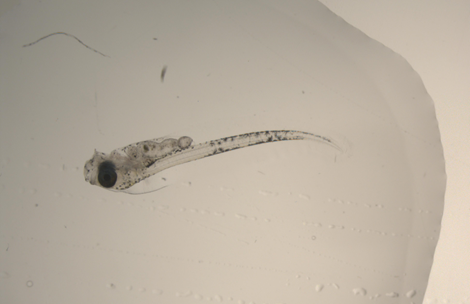


This is a good image. The software can ignore the contrast to the right side of the background as it does not encircle or make contact with the larva. Had it encircled the larva, there is a large likelihood that the segmentation of the larva would have been faulty.


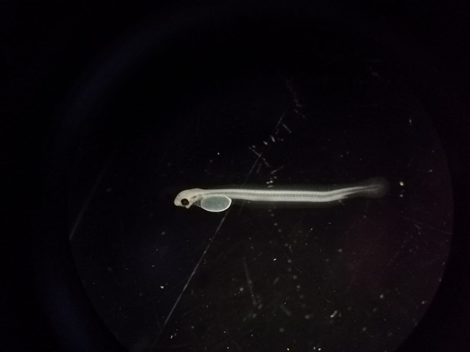


This is a good image. It makes no difference if the background is dark or light.


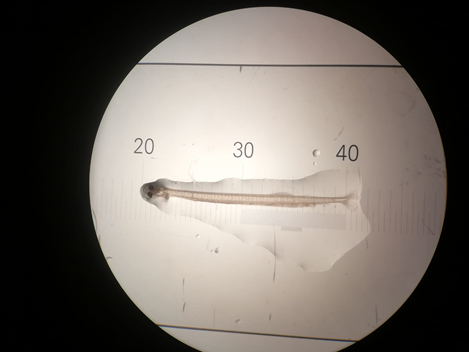


This is a good image. The software includes the option of establishing a rectangular or circular region of interest (ROI) for defining the active area where the larva can be found. In this case the circular ROI would be a good choice.


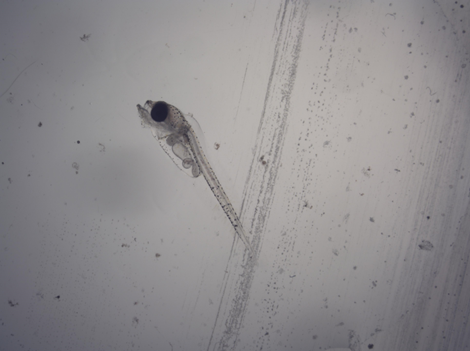


This is a bad image. The strong contrast in the large stripes will be detected and since they are directly associated with the larva, the software will include them as part of the larva.

# Main graphical user-interface overview


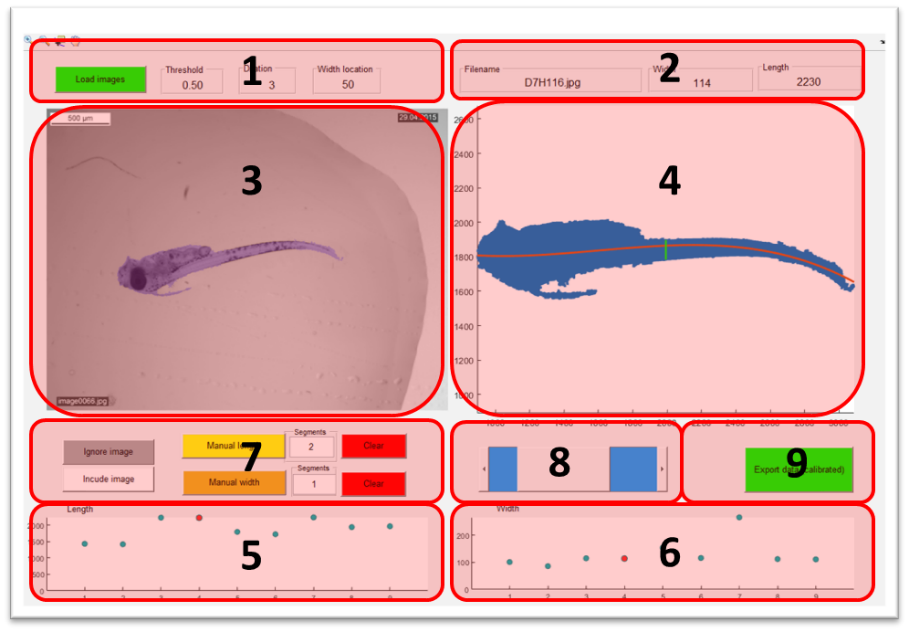


Figure 1

1. Load images and settings panel.
2. Image file information.
3. Original image with segmentation mask overlaid in blue.
4. Zoomed image of segmented larva with indications of locations for automatic length- (red) and depth (green) estimation.
5. Summary panel for length for all images selected.
6. Summary panel for depth for all images selected.
7. Manual measurement panel.
8. Slider for selecting images.
9. Export data button.

# General workflow

1. Press **Load images** button in main GUI to open loading popup.
2. (Optional) Press **Load test image** in loading popup. Select image.
3. In the Calibration panel, press **Create** to generate a calibration of pixels into millimeters or **Retrieve** to use an existing calibration file (this can be done at any point prior to loading.
4. (optional) Test and/or change segmentation settings and set region of interest.
5. **Press Load data** in loading popup to select images for measurement. Loading and processing of images may take some time (around 3 sec per image)
6. Use the slider (panel 8) to review all images to **visually inspect** the automatic segmentation and measurements.
7. Correct faulty automatic measurements by replacing them with **manual length** or **manual depth** measurements. Alternatively, images can be removed from (or included in) the exported data by pressing **Ignore image** (**Include image**).
8. **Export data** as a comma-delimited (csv) file.

Details of these steps are explained in the following sections.

# Loading images


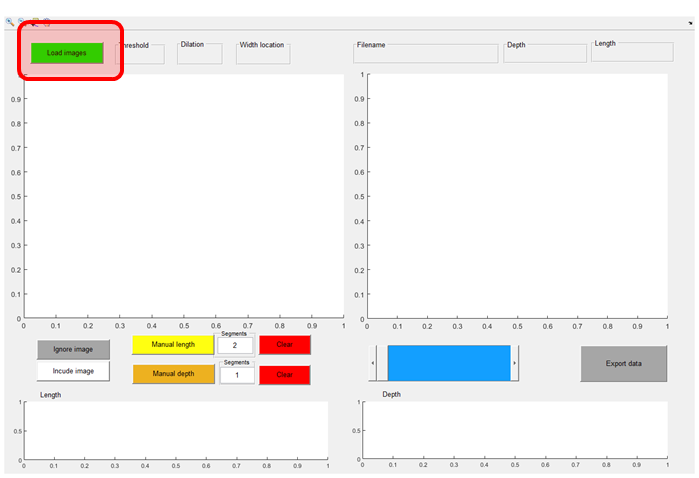


Figure2

Pressing the green **Load images** button in the upper left corner of the main graphical user interface (Figure 2) will open the loading popup window (Figure 3).


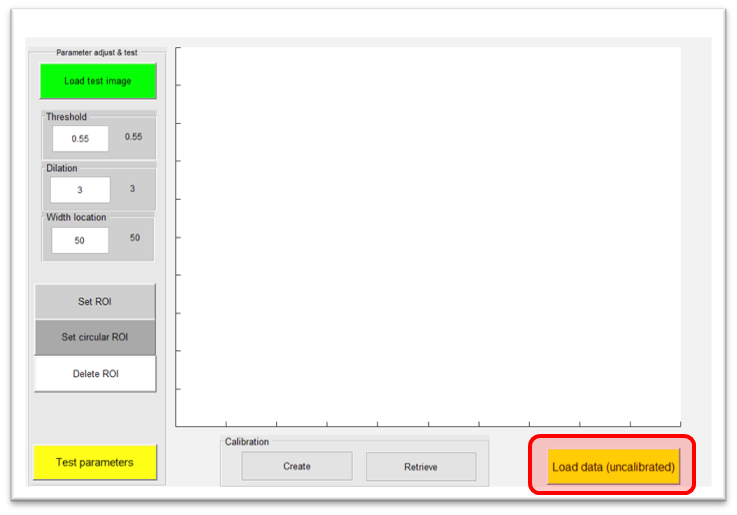


Figure 3

If the default parameters are satisfactory, multiple image files can be loaded and processed using the “**Load data**” button (lower right corner of Figure 3). Standard Windows commands are used for selecting multiple files (shift+ctrl). Calibration into millimeters should be done before loading data.

# Calibration


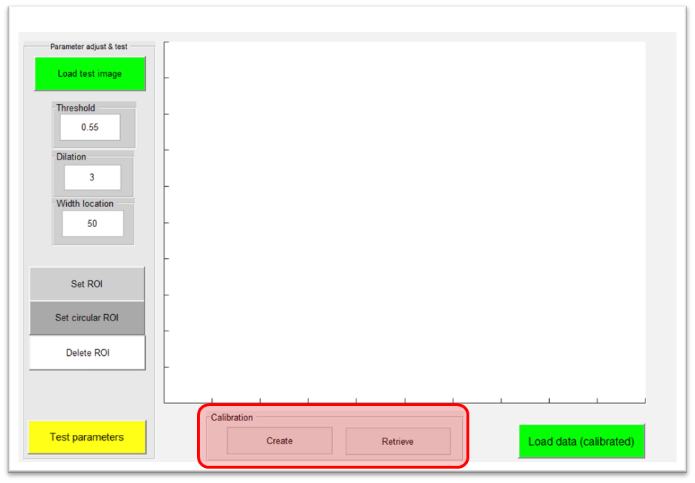


Figure 4

Calibrating the measurements (from pixels to millimeters) can be done in the calibration panel in the loading popup window (shown in red in Figure 4). This panel consists of two buttons. The “**Create**” button opens a dialog box for selecting an image file to be used for calibration. After selecting the image file (which may be the same image uploaded in the previous step), a second graphical user interface is displayed (Figure 5).


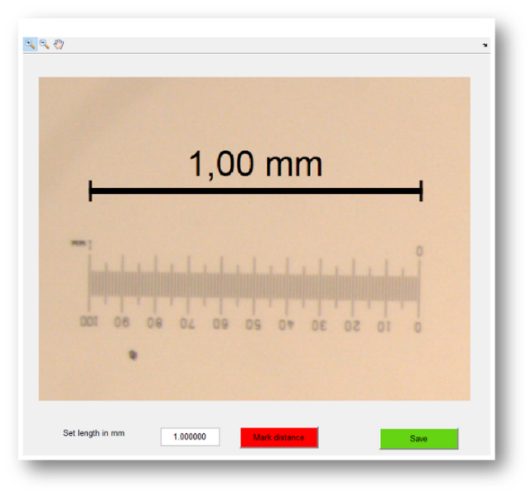


Figure 5

Zoom in on the scale using the zoom tools in the top left of the window (choose the magnifying glass with a plus sign and move the marker to the image to select region to zoom to). Enter the length of the scale in millimeters in the text field (default is 1 mm). Press the “**Mark distance**” button and use the marker to click precisely on one end of the scale followed by the opposite end. Save the calibration via the “**Save**” button. For easy management of calibration files, we suggest keeping “calibrationScale” as the beginning of the name of the saved file and then add a short description of which files the calibration is valid for (e.g., by date, species, treatment, etc.).

After saving the calibration file, the calibration GUI will close and the **Load data** button in the loading popup will change text from “Load data (uncalibrated)” to “Load data (calibrated)” and the color will change from yellow to green.

# Testing parameters for segmentation and setting ROI


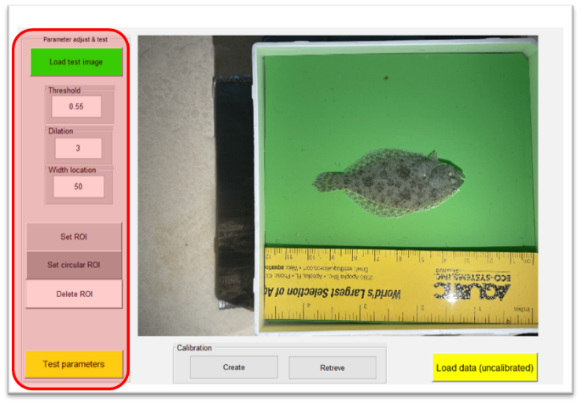


Figure 6

To ensure best possible parameter extraction a representative test image can be loaded, and segmentation settings (threshold and dilation) can be changed and tested before loading in the entire data set (red in Figure 6).

**Threshold** is the amount of contrast compared to the maximum contrast in the image that triggers edge detection. 0.55 has been found to be a good value for most images, but if a trend of too little of the larva is segmented try a lower value. This is especially useful if very dark patches are visible in the ROI selected. The less contrast present in the background, the lower the threshold that should be set. In the example shown in Figure 3, a threshold of around 0.40 gives good segmentation.

**Dilation** is the amount each edge-detected pixel is dilated to create contact with other detected pixels. The default value of 3 should work in most cases, but if a trend of too many protrusions attached to the larval outline is seen, try a lower value. If, on the other hand, a part (e.g., tail) is consistently missed, try a higher value.

Besides the two segmentation settings, the location for extraction of depth can also be set in the loading popup window:

**Depth location** is the percentage of the total length of the larva (measured from the head) at which the depth is measured. To increase robustness of depth measurement, it is not only measured at one point but instead as the median of many measurements made within ±5% of the point selected. Therefore, the depth Location parameter must be between 6 and 94. If a value outside this range is entered, the location is set to the closest valid value (6 or 94). The default value is set to 50 (i.e., measuring depth at the midpoint of the larva).

There is an option to select a region of interest (**ROI**). This is useful in cases where only part of the image should be analyzed. In the example shown in Figure 6, it is beneficial to select a ROI containing only the larva and the green background as the image contains a lot of non-relevant contrast (white box containing fish, yellow measurement scale). Two buttons are available for selecting a ROI: **Set ROI** and **Set circular ROI**. In cases like shown in Figure 6 rectangular ROI is optimal. Procedure is to press the **Set ROI** button, move the marker to the image and mark two diagonal corners of the wanted rectangle by pressing left mouse button. For images with a circular field of view, a circular ROI should be chosen. The set ROI will always be shown in the image and cancelling a selected ROI can be done via the **Delete ROI** button.

# Visual inspection


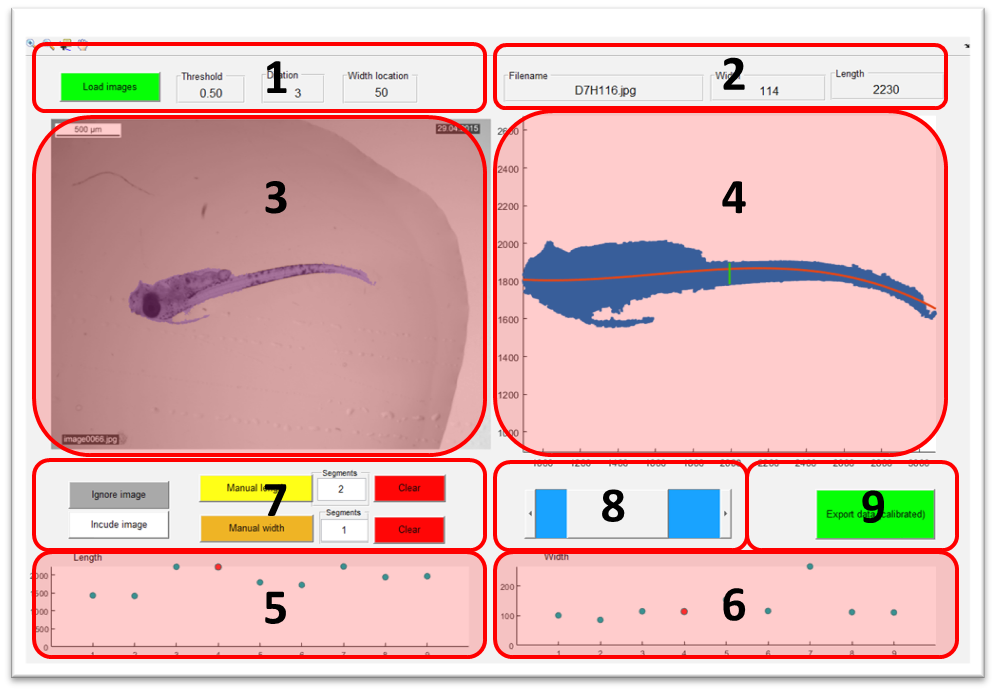


Figure 7

After setting the parameters on the test image, load the images to be measured using the **Load data** button. This will initiate image processing. When this is done, panel 3 can be used to verify correct automatic segmentation. The original image will be shown in grayscale in panel 1 with blue overlay where the segmentation has determined the larva to be present. Notice that if the background contains contrasts, small protrusions on the edges of the larva are unavoidable, but they can be ignored if length and depth are not influenced.

Panel 4 (Figure 7) shows the zoomed-in segmented object with the locations of the length (red line) and depth (green line) measurements to allow for visual detection of errors in these parameters.

Panel 5 (Figure 7) is an overview of the lengths extracted from all images selected for analysis. This facilitates outlier detection. The currently selected image (panels 3 and 4**)** is indicated in red. If automatic length estimation is unsatisfactory and manual measurement of length has been implemented, the marker will change from a circle to a diamond.

Panel 6 is an overview of the depths extracted from all images selected for analysis. This facilitates outlier detection. The selected image (panels 3 and 4) is indicated in red. If automatic depth estimation is unsatisfactory and manual measurement of depth has been implemented, the marker will change from a circle to a diamond.

Navigating between images using the slider (panel 8) or the hotkeys “**a**” and “**s**” to move to the previous and next image in the set, respectively.

Panel 2 supplies information for the currently selected image file. The first field from the left shows the name of the file (i.e. “D7H117.jpg”) and the second field shows the larval depth. The third text field shows the length. If a manual depth length measurement has been done on the selected image, these fields will show the manual measurements, otherwise they will show the automatic measurement.

# Manual measurement


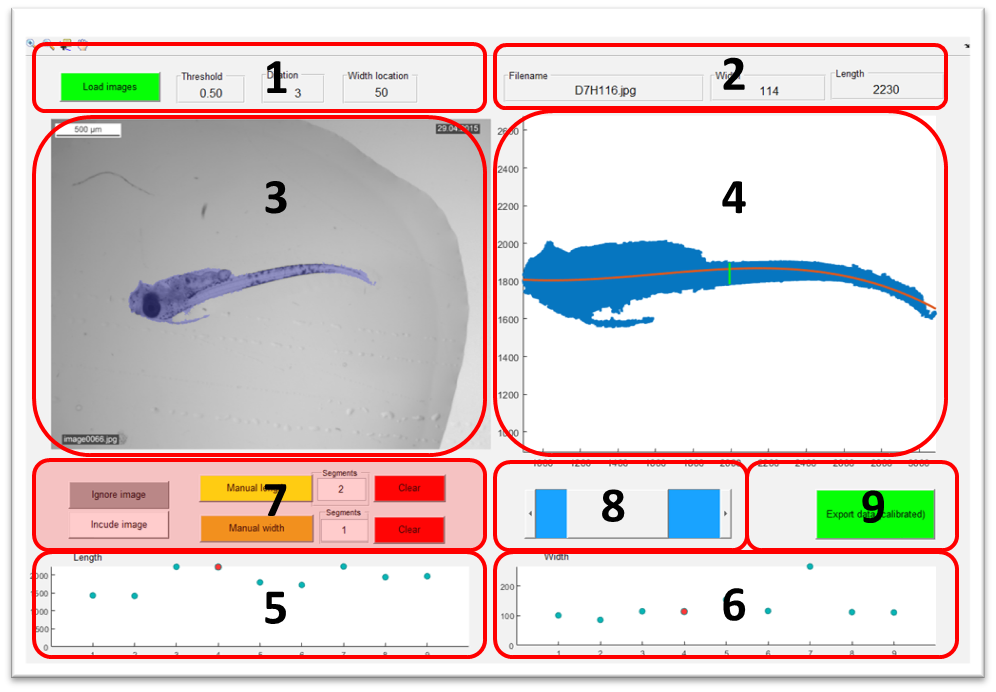


Figure 8

In cases where the automatic parameter extraction is unsatisfactory, manual measurement is easily done using tools in panel 7. The “**Manual length**” button allows for manual length measurement in panel 3. Use the **Segments** field to set the max number of contiguous segments to be used to represent the entire length. The default value is 2 segments. To measure manually, press **Manual length** then click on the image in panel 3 at the beginning of the larva. Then, move the marker to the end of the desired segment and click again. Click again for the end of each succeeding segment. Note that you will need to click one more time than the number of segments you have selected (e.g., three clicks for two segments) in order to finish the measurement, after which the segments will be shown. If not all segments are needed for measuring a specific larva, use of the return button finishes the process. So, if one larva just needs one segment and the length segment is set to 3 just marking the two ends of the segment and finishing with “return” is possible. To revert to the automatic measurement after having entered a manual measurement, click the “**Clear**” button to the right.

The “M**anual depth**” button works in the same way as the **Manual length** button, but as most depths can be drawn by using only one segment, the default setting for the **Segments** field is 1, but this can be changed.

The two buttons **Ignore image** and **Include image** are for cases where the image is not good enough to extract accurate measurements. **Ignore image** deletes the image from the data set. It also removes the length and depth measurements from panels 5 and 6. If an image has been ignored by mistake, pressing **Include image** will restore the image and its data will be included in the data set and the two summary windows.

# Export data


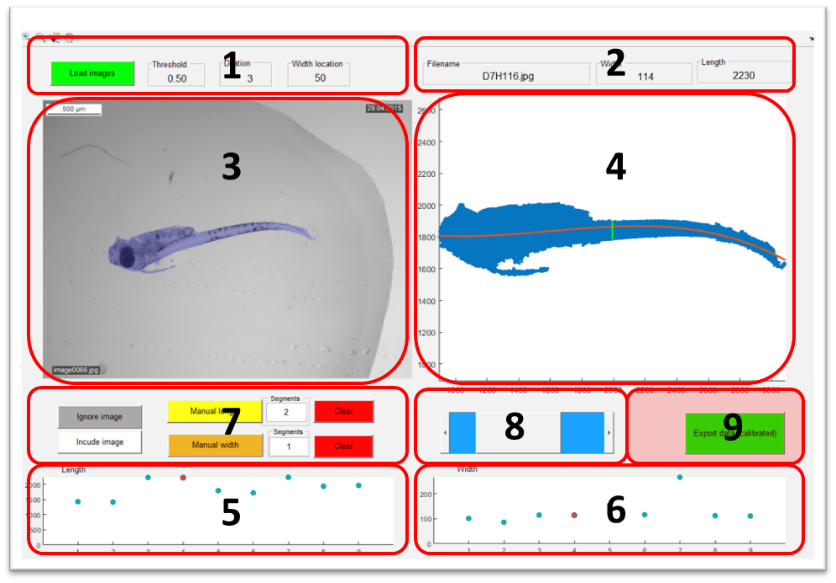


Figure 9

Exporting data is done via the “**Export data**” button (Panel 9 marked red in Figure 9). If no calibration has been applied to the data, the button is yellow to remind the user to calibrate the data before exporting. When a calibration file has been loaded, the **Export data** button is green.

When the button is pressed, a suggested file name appears which contains all relevant information about parameter extraction. We suggest appending a short description to this suggested file name.

The output is a CSV file with a row for each image file and columns of data (Table 1)

Columns 6-9 (red in Table 1) are only present if the data set has been calibrated.

Pixel based measurements (columns 1-5) are included in the output in case calibration into millimeters is not possible.

LengthAutomated and DepthAutomated are included in the output file for eventual analysis of how well the automatic parameter segmentation works.

Accuracy of length and depth measurements can never be better than one pixel and hence the calibrated output is rounded to significant digits that reflect the size of the pixels.
